# Substrate structure determines p97- and RAD23A/B-mediated proteasomal degradation in human cells

**DOI:** 10.1093/jb/mvaf046

**Published:** 2025-08-11

**Authors:** Yi Ding, Takuya Tomita, Hikaru Tsuchiya, Yasushi Saeki

**Affiliations:** Division of Protein Metabolism, The Institute of Medical Science, The University of Tokyo, 4-6-1 Shirokanedai, Minato-ku, Tokyo 108-8639, Japan; Division of Protein Metabolism, The Institute of Medical Science, The University of Tokyo, 4-6-1 Shirokanedai, Minato-ku, Tokyo 108-8639, Japan; Department of Physiology, Graduate School of Medicine, Juntendo University, 2-1-1 Hongo, Bunkyo-ku, Tokyo 113-8421, Japan; Autophagy Research Center, Juntendo University Graduate School of Medicine, 2-1-1 Hongo, Bunkyo-ku, Tokyo 113-8421, Japan; Division of Protein Metabolism, The Institute of Medical Science, The University of Tokyo, 4-6-1 Shirokanedai, Minato-ku, Tokyo 108-8639, Japan

**Keywords:** p97, RAD23A/B, substrate structure, ubiquitin-fusion degradation, ubiquitin-proteasome system

## Abstract

Proteasomal degradation of ubiquitinated proteins involves various accessory factors, including p97 and shuttle factors, but their requirements and relationship with substrate structural properties are not fully understood, especially in human cells. Here, we demonstrate that substrate structure dictates the dependency on p97 and RAD23A/B for proteasomal degradation in human cells, using two ubiquitin-fusion model substrates, Ub-GFP (well-folded) and Ub-GFP-tail (with an unstructured tail). Both substrates exhibited similar ubiquitin chain composition, primarily mediated by the UBR4–KCMF1 E3 ligase. Interactome analyses revealed that Ub-GFP preferentially interacts with p97 and RAD23B, while Ub-GFP-tail binds more strongly with the proteasome. The degradation of Ub-GFP depends on p97 and RAD23A/B, whereas that of Ub-GFP-tail bypasses these accessory factors. RAD23A/B knockdown resulted in a reduction in the apparent lengths of ubiquitin chains on both substrates, yet it only affected Ub-GFP degradation, suggesting that even a lower level of ubiquitination is sufficient to support proteasomal degradation of substrates with an unstructured tail. Overall, our findings highlight substrate structure as a key determinant of accessory factor requirement, offering valuable insights for the development of targeted protein degradation.

## Graphical Abstract

**Graphical Abstract:**
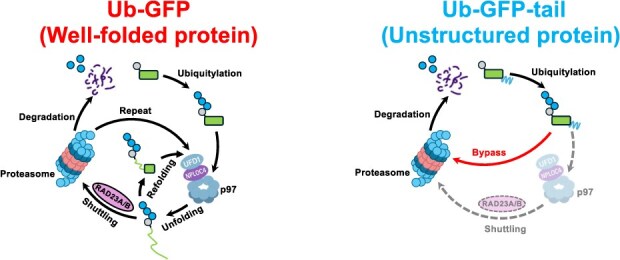

## Abbreviation

AQUA: absolute quantification
CHX: cycloheximide
DDA: data-dependent acquisition
DIA: data-independent acquisitions
E1: ubiquitin activating enzyme
E2: ubiquitin conjugating enzyme
E3: ubiquitin ligase
ERAD: endoplasmic reticulum-associated degradation
IAA: iodoacetamide
IP: immunoprecipitation
MS: mass spectrometry
PRM: parallel reaction monitoring
PROTAC: proteolysis-targeting chimera
RIPA: radioimmunoprecipitation assay
TEAB: triethylammonium bicarbonate
TFA: trifluoroacetic acid
Ub: ubiquitin
UBA: ubiquitin-associated
UBL: ubiquitin-like
UFD: ubiquitin-fusion degradation
UPS: ubiquitin-proteasome system
WB: Western blot

The ubiquitin-proteasome system (UPS) is the primary pathway for protein degradation in eukaryotic cells, responsible for the turnover of most intracellular proteins *(**1**)*. By selectively degrading proteins, the UPS maintains cellular homeostasis and regulates various biological processes, including cell cycle progression, transcriptional regulation and protein quality control *(**2*–*5**)*. Target protein substrates of the UPS are typically tagged by the covalent attachment of multiple ubiquitin molecules, forming a polyubiquitin chain. This tagging mechanism enables the cell to distinguish specific proteins for degradation from the vast pool of cellular proteins. Ubiquitination is carried out by an enzymatic cascade involving E1 activating enzymes, E2 conjugating enzymes and E3 ubiquitin ligases *(**6**,*  *7**)*. In particular, E3 ligases ubiquitinate target substrates by recognizing specific degron motifs or post-translational modifications and catalysing ubiquitin transfer from an E2 enzyme. Through successive rounds of ubiquitination, a polyubiquitin chain is built on the substrate, usually linked via lysine (K)-48 residues of ubiquitin, to form the proteasomal degradation signal. Once polyubiquitinated, the substrate is recognized and processed by the 26S proteasome, a large ATP-dependent protease complex that executes protein degradation. Although K48-linked chains are generally considered the primary signal for proteasomal degradation, recent studies have revealed that branched ubiquitin chains—such as K11/K48, K29/K48 and K63/K48 branches—can enhance the ubiquitin signal and serve as robust signal for proteasomal degradation *(**8*–*12**)*. Additionally, *in vitro* studies have established that the degradation of ubiquitinated substrates by the purified 26S proteasome requires an unstructured region, termed the ‘initiation site’, where the proteasome can directly engage *(**13*–*16**)*.

The UPS is not a simple linear pathway but a highly regulated and dynamic network that integrates multiple factors to ensure precise control of protein degradation *(**2**,*  *17**)*. This network involves a coordinated interplay of various ubiquitin enzymes and accessory factors, including E3 ligases, deubiquitinases, ubiquitin-shuttle factors and the AAA+ ATPase p97. Among these, p97 (Cdc48 in yeast) and ubiquitin-shuttle proteins like RAD23A/B are particularly important for unfolding substrates and delivering them to the proteasome.

p97 is a homohexameric AAA+ ATPase that functions as a segregase/unfoldase upstream of the proteasome. It converts the energy of ATP hydrolysis into mechanical force to extract ubiquitinated proteins from complexes or membranes and unfold them *(**18**,*  *19**)*. In the context of protein degradation, p97 and its cofactors recognize polyubiquitinated substrates that the proteasome cannot easily handle on its own—often because these substrates are tightly folded or trapped within protein complexes—and then extract these substrates and unfold them. Through these processes, p97 prepares otherwise hard-to-degrade proteins for subsequent destruction by the proteasome. This unfoldase activity is critical in processes like endoplasmic reticulum-associated degradation (ERAD) and in the clearance of protein aggregates *(**20**,*  *21**)*. Furthermore, its unfolding activity is essential for certain cytosolic/nuclear substrates that lack accessible unstructured regions *(**22**)*.

Shuttle factors are proteins that contain dual domains—a ubiquitin-like (UBL) domain that binds to the proteasome and one or more ubiquitin-associated (UBA) domains that bind to polyubiquitin chains on substrates. Essentially, they act as adaptor molecules, capturing ubiquitinated proteins and ‘shuttling’ them to the proteasome for degradation *(**3**,*  *17**)*. Budding yeast has two major shuttle factors, Rad23 and Dsk2, which are known to be involved in the ubiquitin-fusion degradation (UFD), cell cycle progression and ERAD pathways and are largely functionally redundant *(**23*–*25**)*. Importantly, Rad23 and Dsk2 function cooperatively with Cdc48, thereby processing most ubiquitinated substrates within yeast cells *(**24*–*27**)*. In human cells, the shuttle factors exhibit greater diversity than yeast, including RAD23A, RAD23B and UBQLN1–4 *(**17**)*. The exact functions and mechanisms of those shuttle factors are less characterized. RAD23A/B have been shown to have high affinity for K48-linked ubiquitin chains, are widely expressed and bind a broad range of ubiquitinated proteins in cells *(**17**,*  *23**,*  *28**)*. Previous studies have demonstrated that the UBL domain of RAD23A/B can activate the proteasome and facilitate substrate degradation *(**29**,*  *30**)*. Moreover, studies in human cells have demonstrated that RAD23B, through multivalent interactions with polyubiquitin chains, forms condensates with p97, polyubiquitinated substrates and the 26S proteasome, which function as sites for protein degradation *(**31*–*33**)*. Nevertheless, the detailed function of human RAD23A/B remains to be determined.

UFD substrates consist of an uncleavable ubiquitin moiety (*e.g.* Ub-G76V) fused to a reporter protein, which led to the groundbreaking identification of a series of important regulators in the UFD pathway in yeast, including Ufd1–5, Cdc48, Rad23 and Dsk2 *(**25**,*  *34*–*36**)*. Ub-GFP is a short-lived soluble model UFD substrate that has been widely used to monitor proteasome activity in yeast, human cells and animals such as mice and nematodes *(**35**,*  *37**,*  *38**)*. Despite the identification of homologous genes for the yeast UFD pathway in humans and mice, the precise molecular mechanisms by which Ub-GFP is degraded in these higher eukaryotes remain to be fully elucidated. Also, a previous study in yeast demonstrated that Ub-GFP fused to a 20-amino-acid unstructured sequence can be degraded by the proteasome without ubiquitin chain formation, Cdc48 or Rad23 *(**39**)*, but this bypass mechanism in human cells remains unclear.

In this study, we investigated how the unstructured region of substrate proteins affects proteasomal degradation pathways. We systematically compared two UFD substrates, Ub-GFP and Ub-GFP-tail, regarding their ubiquitin chain types, responsible E3 ligases and requirements for p97 and RAD23A/B for degradation. We found that both Ub-GFP and Ub-GFP-tail were modified with K29/K48-linked ubiquitin chains, primarily mediated by the UBR4–KCMF1 E3 ligase. While Ub-GFP degradation requires p97 and RAD23A/B, Ub-GFP-tail bypasses these factors. Despite RAD23A/B knockdown leading to shorter ubiquitin chain lengths on both substrates, Ub-GFP-tail degradation remained unaffected, suggesting that short ubiquitin chains are sufficient for proteasomal degradation of substrates with an unstructured tail. Our findings demonstrate that substrate structure dictates the proteasomal degradation pathway in human cells, revealing that well-folded substrates strongly depend on p97 and RAD23A/B, whereas substrates with accessible unstructured tail bypass these requirements.

## Materials and Methods

### Cell culture

HCT116 cells were obtained from the American Type Culture Collection and maintained (37°C, 5% CO_2_) in DMEM high-glucose medium (Nacalai Tesque), supplemented with 10% foetal bovine serum (Thermo Fisher Scientific), penicillin–streptomycin (100 U/ml, Gibco) and MEM nonessential amino acids (1×, Gibco).

### Generation of stable HCT116 cell lines

To generate HCT116 cell lines stably expressing Ub-G76V-GFP or Ub-G76V-GFP-tail, we constructed two Tol2-based expression plasmids using the pAID6.3N backbone (modified from pAID6.3 by removing the AID cassette and retaining the Tol2 elements) *(**40**,*  *41**)*. The Ub-G76V-GFP coding sequence (Addgene #11941) or Ub-G76V-GFP fused with a 65-amino-acid sequence derived from cyclin B containing an ssrA tag (sequence: AANDENYALAAHGGKHTFNNENVSARLGGACSIAVQAPAQHTFNNENVSARLGGALSIAVQAPAQ) *(**42**)* was inserted into the pAID6.3N. An IRES-mCherry cassette was also added downstream as an internal expression control. HCT116 cells were co-transfected with each construct and the pCS-TP transposase expression vector using Lipofectamine 3000 (Thermo Fisher Scientific) *(**41**)*. At 24 h post-transfection, 100 μg/ml hygromycin B (Thermo Fisher Scientific) was added for selection. Hygromycin-resistant clones were isolated and validated by Western blot and confocal fluorescent imaging analysis using a CellVoyager CQ1 (Yokogawa).

### RNA interference

siRNA oligos were obtained from ON-TARGETplus SMARTpool (Dharmacon). The siRNAs utilized were pools of four different sequences as follows: RAD23A siRNAs: L-005231-00, RAD23B siRNAs: L-005231-00, UBR4 siRNAs: L-014021-01, KCMF1 siRNAs: L-017900-01, UBE4B siRNAs: L-007202-00, TRIP12 siRNAs: L-007182-00. For non-targeting control siRNA, we used ON-TARGETplus non-targeted siRNA#3 (Dharmacon, L-011365-00). siRNAs were transfected into cells using Lipofectamine RNAiMAX (Thermo Fisher Scientific). After 24 h of transfection, the medium was replaced, and the cells were grown for an additional 48 h before analysis.

### Inhibitor treatment

Bortezomib (BTZ) (Wako), NMS-873 (Sigma-Aldrich), TAK-243 (Selleck Chemicals) and cycloheximide (CHX) (Wako) were purchased from the indicated manufacturers. For substrate stability assays, cells were treated with the indicated inhibitors for 3 h prior to cell lysis. For CHX chase analysis, cells were transfected with siRNAs targeting RAD23A/B for 72 h, followed by treatment with 50 μg/ml of CHX for 0, 15, 30 or 60 min before cell lysis.

### Immunoblotting

Cells were lysed with buffer A (50 mM Tris–HCl, pH 7.5, 100 mM NaCl, 10% glycerol, 10 mM iodoacetamide (IAA) (Sigma-Aldrich)) containing 0.2% NP-40, 10 μM BTZ, 10 μM PR619 (LifeSensors) and complete protease inhibitor cocktail (Roche, EDTA free) for 30 min on ice, and then centrifuged at 12,000 × *g* for 5 min at 4°C. Supernatants were collected, and protein concentration was measured using the BCA protein assay kit (Thermo Fisher Scientific). Cell lysates were boiled in 1× LDS NuPAGE sample buffer for 10 min, and then electrophoresed on 4–12% NuPAGE Bis-Tris gels (Thermo Fisher Scientific). Proteins were transferred to polyvinylidene difluoride membranes (Millipore). The membranes were blocked for 30 min in 5% non-fat milk, and then incubated for 1 h at room temperature with primary antibodies. The primary antibodies used were as follows: anti-GFP mouse monoclonal (clones 7.1 and 13.1, Roche), anti-ubiquitin HRP-conjugated mouse monoclonal (sc-8017; Santa Cruz Biotechnology) and anti-mCherry rabbit polyclonal (Sigma-Aldrich). After extensive washing with TBST, the membranes were incubated with secondary antibodies for 30 min at room temperature. The following secondary antibodies were purchased from Promega: HRP-conjugated goat anti-rabbit IgG and HRP-conjugated goat anti-mouse IgG. After washing several times with TBST, blots were developed using ECL Prime Western Blotting Detection Reagent (Cytiva) and analysed on a Fusion FX7 (Vilber Bio Imaging). Acquired images were quantified using ImageJ.

### Immunoprecipitation using GFP-Trap

For interactome analysis, cells were collected and lysed in buffer A containing 0.2% NP-40, 10 μM BTZ, 10 μM PR619 (LifeSensors) and complete protease inhibitor cocktail (Roche, EDTA free). For denaturing immunoprecipitation (IP) to monitor ubiquitination, cells were lysed in radioimmunoprecipitation assay (RIPA) buffer (50 mM Tris–HCl, pH 8.0, 0.1% SDS, 150 mM NaCl, 1% NP-40, 0.5% sodium deoxycholate) containing a complete protease inhibitor cocktail, 10 μM BTZ and 10 mM IAA. After incubation on ice for 30 min, the lysate was sonicated using a Handy Sonic (ACTIVE MOTIF) and then clarified by centrifugation at 20,000 × *g* for 15 min at 4°C. Protein concentration was determined using the BCA protein assay kit (Thermo Fisher Scientific). For anti-GFP IP, 1 mg of cell lysate was incubated with GFP-Trap Agarose beads (Proteintech) for 1 h at 4°C. After washing three times with either buffer A containing 0.2% NP-40 or RIPA buffer (for denaturing IP), proteins were then eluted by incubating the beads for 5 min at 95°C in 1× NuPAGE LDS sample buffer.

### Absolute quantification of ubiquitin linkages (Ub-AQUA/PRM)

For mass spectrometry (MS)-based absolute quantification of the ubiquitin chains (Ub-AQUA/PRM) *(**43**)*, immunoprecipitated proteins, denatured in 1× NuPAGE LDS sample buffer, were subjected to trypsin digestion using the SP3 protocol on a KingFisher APEX system. Specifically, for SP3-based digestion, magnetic carboxylate-modified beads (Cytiva Sera-Mag SpeedBead Carboxylate-Modified Magnetic Particles, hydrophilic and hydrophobic, mixed 1:1) were used. Proteins were first reduced and alkylated using 10 mM dithiothreitol (10 min, 70°C) and 15 mM IAA (15 min, room temperature, dark), followed by binding to beads in 50% ethanol. The KingFisher APEX was programmed to perform sequential washing (3× with 80% ethanol) and digestion (37°C, 3.5 h) in 100 μl of triethylammonium bicarbonate (TEAB) buffer containing 0.5 μg Trypsin Gold (MS grade, dissolved in 50 mM TEAB; Thermo Fisher Scientific). After digestion, peptides were eluted with 100 μl MS-grade water and acidified with 0.1% trifluoroacetic acid (TFA). Then peptides were spiked with Ub-AQUA peptides (25 fmol/injection), concentrated using a speed-vac and then diluted with 0.1% TFA and 0.05% H_2_O_2_ to oxidize Met. Samples were then analysed by connecting an Easy-nLC 1200 (Thermo Fisher Scientific) to an Orbitrap Fusion Lumos Tribrid mass spectrometer (Thermo Fisher Scientific) equipped with a Nanospray Flex Ion Source (Thermo Fisher Scientific). Peptides were separated on a C18 analytical column (3-μm particle size, 75-μm diameter × 120-mm length, Nikkyo Technos #NTCC-360/75-3-125), using a 60-min gradient of acetonitrile at a flow rate of 300 nl/min (0–55 min: acetonitrile from 0% to 28%, 55–57 min: acetonitrile from 32% to 80%, and 57–60 min: maintained at 80%). Targeted acquisition of MS/MS spectra was performed in the parallel reaction monitoring (PRM) mode, with an orbitrap resolution of 15,000, isolation window of 2.0 *m*/*z* and fragmentation by HCD with normalized collision energies of 28, using the Xcalibur software 2.2. Data were analysed using the PinPoint 1.3 software (Thermo Fisher Scientific).

### MS-based proteome analysis

Cell lysates or immunoprecipitates, denatured in 1× NuPAGE LDS sample buffer, were subjected to trypsin digestion using the SP3 protocol on a KingFisher APEX system, as described above. After digestion, samples were centrifuged at 15,000 rpm for 15 min at 4°C, and the supernatants were collected.

For proteome analysis shown in Fig. 3B, desalting was performed using Evotip pure columns (Evosep), involving equilibration with Evosep buffer B (0.1% formic acid in acetonitrile), sample loading and washing with Evosep buffer A (0.1% formic acid). Finally, samples were separated on a C18 analytical column (1.7-μm particle size, 75-μm diameter × 150-mm length, IonOpticks #3-15075C18-5) using an Evosep One liquid chromatography system (Evosep) with the Whisper Zoom 20SPD programme, connected to an Orbitrap Fusion Lumos Tribrid mass spectrometer (Thermo Fisher Scientific) equipped with a Dream Spray ING ion source (AMR). Data were acquired using a data-independent acquisition (DIA) method with FAIMS-pro set to −45 CV. Precursor MS spectra were collected in the range of 484–868 *m/z* (isolation window: 8 *m/z*, window overlap: 1 *m/z*). MS/MS spectra were collected in the range of 200–1,800 *m/z* at a resolution of 50,000. The HCD was set to 28. Raw files were analysed using Proteome Discoverer 3.2 software (Thermo Fisher Scientific) with the Chimerys search engine against the *Homo sapiens* reference proteome (UniProt, version 2024-10-02). The mass tolerance for the fragment ions was set to 20 ppm. Methionine oxidation and diglycine–lysine were set as variable modifications, and cysteine carbamidomethylation was set as a static modification for database searching. Maximum missed cleavage sites of trypsin were set to 2. Peptide identification was filtered at a false discovery rate (FDR) <0.01. Label-free, intensity-based protein quantification was performed using the Fragment Ions Quantifier node in Proteome Discoverer 3.2. Protein abundances were normalized according to the total peptide amounts.

For the quantification of GFP used for normalization in Fig. 3E and F, desalting was performed using Evotip pure columns (Evosep), as described above. Samples were then separated on a C18 analytical column (1.7-μm particle size, 75-μm diameter × 150-mm length, IonOpticks #3-15075C18-5) using an Evosep One liquid chromatography system (Evosep) with the Whisper Zoom 40SPD programme, connected to an Orbitrap Fusion Lumos Tribrid mass spectrometer (Thermo Fisher Scientific) equipped with a Dream Spray ING ion source (AMR). Data were acquired using a data-dependent acquisition (DDA) method with a mass range of 350–2,000 *m*/*z* and a resolution of 500,000 using the Xcalibur software 2.2. The HCD was set to 30 with a 3-s cycle selecting the most intense ions. MS raw files were analysed using Proteome Discoverer 3.2 software (Thermo Fisher Scientific) with the Sequest HT search engine against the *H. sapiens* reference proteome (UniProt, version 2024-10-02) appended with an EGFP sequence. The precursor and fragment mass tolerances were set to 10 ppm and 0.02 Da, respectively. Methionine oxidation, protein amino-terminal acetylation, diglycine–lysine and glutamine/asparagine deamidation were set as variable modifications, and cysteine carbamidomethylation was set as a static modification for database searching. Maximum missed cleavage sites of trypsin were set to 2. Peptide identification was filtered at false discovery rate <0.01. Label-free, intensity-based protein quantification was performed using the Precursor Ions Quantifier node in Proteome Discoverer 3.2.

For interactome analysis shown in Fig. 4B, desalting was performed using GL-Tip SDB, involving equilibration with buffer B (80% acetonitrile, 0.1% TFA), sample loading and washing with buffer A (0.1% TFA). Samples were then analysed by connecting an Easy-nLC 1200 to an Orbitrap Fusion Lumos Tribrid mass spectrometer equipped with a Nanospray Flex Ion Source. Peptides were separated on a C18 analytical column (3-μm particle size, 75-μm diameter × 120-mm length, Nikkyo Technos #NTCC-360/75-3-125), using a 75-min gradient of acetonitrile at a flow rate of 300 nl/min (0–60 min: acetonitrile from 0% to 0%, 60–70 min: acetonitrile from 40% to 80%, and 70–75 min: maintained at 80%). Data were acquired using a DDA method and analysed using Proteome Discoverer 3.2 software (Thermo Fisher Scientific), as described above.

**Fig. 1:**
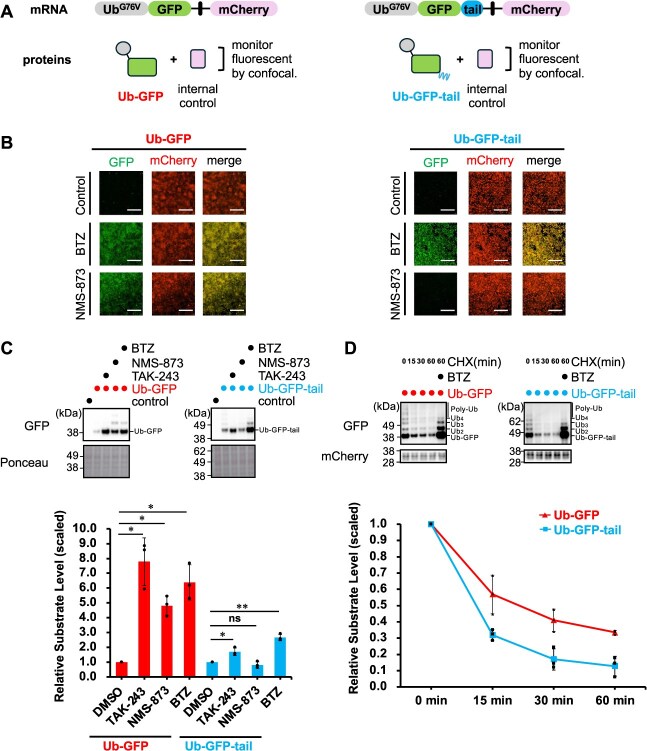
**Ub-GFP and Ub-GFP-tail exhibit different dependencies on p97 for UPS-mediated degradation.** (A) Schematic of two UFD model substrates. Both constructs contain an N-terminal G76V-mutant ubiquitin (Ub^G76V^) fused to GFP, followed by an IRES-linked mCherry for internal expression control. Ub-GFP-tail additionally carries a 65-amino-acid unstructured cyclin B tail fused to the C-terminus of GFP. (B) Confocal imaging of HCT116 cells expressing Ub-GFP (left) or Ub-GFP-tail (right) after treatment with DMSO, proteasome inhibitor BTZ (1 μM, 3 h) or p97 inhibitor NMS-873 (10 μM, 3 h). Scale bars, 500 μm. (C) WB analysis of Ub-GFP and Ub-GFP-tail substrate levels after treatment with DMSO, E1 inhibitor TAK-243 (1 μM, 3 h), BTZ or NMS-873. Ponceau staining is shown as a loading control. Quantification of substrate levels normalized to the matched DMSO control is shown below (mean ± SEM, *n* = 3). Statistical significance was determined using a two-tailed one-sample *t*-test (^*^P < 0.05, ^**^P < 0.01, ^***^P < 0.001; ns, not significant). (D) CHX chase assay of Ub-GFP and Ub-GFP-tail. HCT116 cells were treated with CHX, and substrate levels were assessed at the indicated time points. Quantification of GFP levels normalized to time 0 is shown below (mean ± SD, *n* = 3).

## Results

### Substrate structure dictates p97-dependency in human cells

First, we established two HCT116 cell lines stably expressing UFD model substrates: Ub^G76V^-GFP (Ub-GFP) and Ub^G76V^-GFP-cyclin B tail (Ub-GFP-tail) (Fig. 1A). The G76V mutation of ubiquitin (Ub) prevents deubiquitinase-mediated removal of the initial Ub, thereby mimicking monoubiquitinated substrates. The Ub-GFP-tail substrate includes a 65-amino-acid unstructured cyclin B sequence at the C-terminus of GFP *(**42**)*. For internal expression control, an mCherry reporter was fused via an IRES sequence downstream of both Ub-GFP and Ub-GFP-tail constructs. Confocal fluorescent imaging and Western blot (WB) analysis confirmed the successful generation of both cell lines (Fig. 1B and C). As expected, treatment with the proteasome inhibitor BTZ or the E1 inhibitor (TAK-243) caused accumulation of both substrates, demonstrating that they are degraded via the UPS. However, the p97 inhibitor (NMS-873) caused accumulation of only Ub-GFP, not Ub-GFP-tail (Fig. 1B and C, Table S1A). This observation strikingly demonstrates for the first time in human cells that Ub-GFP degradation requires p97-mediated activity (likely unfolding), whereas the unstructured tail on Ub-GFP-tail bypasses this requirement, consistent with previous studies in yeast *(**39**,*  *42**)*. To further confirm the rapid proteasomal turnover of both substrates, we performed CHX chase experiments, which revealed rapid degradation of both substrates, with Ub-GFP-tail exhibiting a faster degradation rate than Ub-GFP (half-lives: Ub-GFP, ~17 min; Ub-GFP-tail, ~12 min) (Fig. 1D and Table S1B).

### Both Ub-GFP and Ub-GFP-tail are ubiquitinated with K29/K48-linked chains

**Fig. 2:**
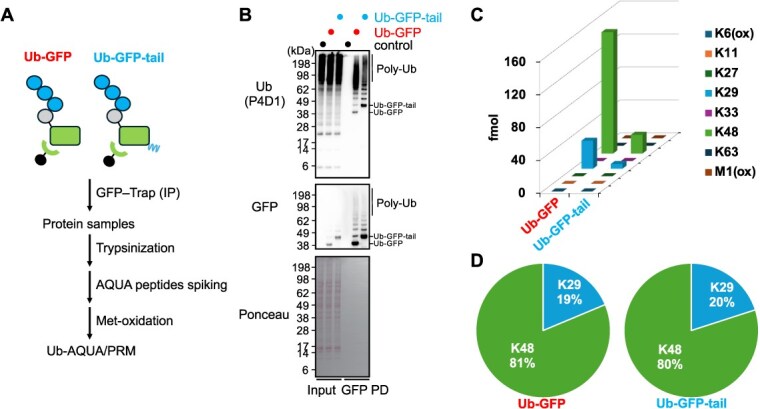
**Ub-GFP and Ub-GFP-tail exhibit similar basal ubiquitin-chain composition.** (A) Schematic workflow of Ub-AQUA/PRM analysis. (B) WB analysis of whole-cell lysates (Lanes 1–3) and GFP immunoprecipitates (Lanes 4–6) from HCT116 cells expressing Ub-GFP or Ub-GFP-tail. Blots were probed with anti-ubiquitin (P4D1) and anti-GFP antibodies. (C) Absolute quantification of ubiquitin chain linkages on GFP-immunoprecipitated substrates using Ub-AQUA/PRM. *n* = 2, biological replicates. (D) Ubiquitin-chain composition of GFP-purified substrates determined by Ub-AQUA/PRM.

To understand the underlying causes for the observed difference in degradation pathways between these two substrates, we next investigated their ubiquitination profiles. We performed GFP pull-down followed by MS-based absolute quantification of ubiquitin linkages via Ub-AQUA/PRM (Fig. 2A). WB analysis demonstrated that both Ub-GFP and Ub-GFP-tail are highly polyubiquitinated even under steady-state conditions (Fig. 2B). Subsequent MS analysis further revealed that both substrates are predominantly modified with K48-linked ubiquitin chains (Fig. 2C and D, Table S2A). While WB indicated subtle differences in their overall ubiquitination patterns, quantitative MS analysis of Ub linkages suggested similar relative proportions of K48 and K29 linkages on both substrates (approximately 80% K48, 20% K29). Therefore, we next sought to determine the specific E3 ubiquitin ligases responsible for ubiquitinating these UFD substrates.

### UBR4–KCMF1 mediates ubiquitination of both Ub-GFP and Ub-GFP-tail

**Fig. 3:**
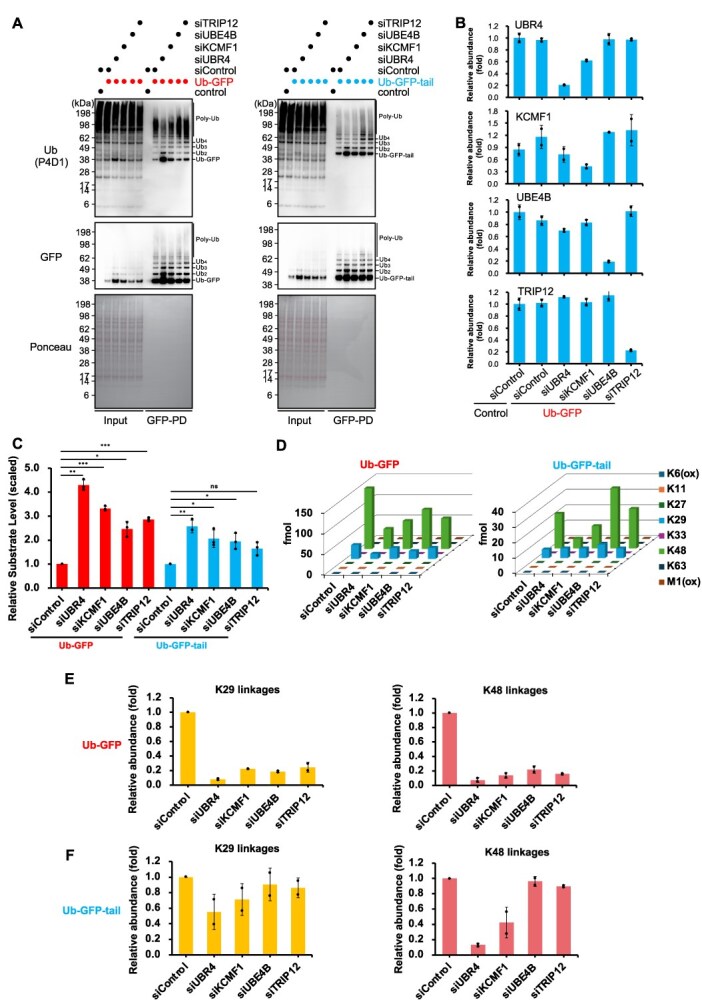
**E3 ligases regulate the ubiquitination of Ub-GFP and Ub-GFP-tail.** (A) WB analysis of whole-cell lysates (Lanes 1–6) and GFP immunoprecipitates (Lanes 7–12) from HCT116 cells expressing Ub-GFP (left) or Ub-GFP-tail (right) following transfection with siRNAs targeting UBR4, KCMF1, UBE4B, TRIP12 or a control siRNA. (B) Relative expression levels of each E3 ligase in whole-cell lysates as determined by DIA-MS, confirming siRNA-mediated knockdown efficiency. Protein abundances were normalized to the average value of the siControl condition. (C) Quantification of Ub-GFP and Ub-GFP-tail levels in whole-cell lysates, normalized to the matched siControl (mean ± SEM, *n* = 3). Statistical significance was determined using a two-tailed one-sample *t*-test (^*^P < 0.05, ^**^P < 0.01, ^***^P < 0.001; ns, not significant). (D) Absolute quantification of ubiquitin chain linkages on GFP-purified substrates in different siRNA conditions, determined by Ub-AQUA/PRM. *n* = 2, biological replicates. (E) Relative abundance of K29- and K48-linked ubiquitin chains on Ub-GFP in each siRNA condition, determined by Ub-AQUA/PRM. Peptide abundances were normalized to the corresponding substrate levels and scaled to the siControl condition (mean ± SEM, *n* = 2). (F) Relative abundance of K29- and K48-linked ubiquitin chains on Ub-GFP-tail in each siRNA condition, determined by Ub-AQUA/PRM. Peptide abundances were normalized to the corresponding substrate levels and scaled to the siControl condition (mean ± SEM, *n* = 2).

Previous studies have identified several E3 ubiquitin ligases that target UFD substrates, including TRIP12, HUWE1, UBR4 and KCMF1 *(**44*–*48**)*. In addition, UBE4B, a yeast Ufd2 homologue, and the ubiquitin-binding E3 ubiquitin ligase UBR5 have been suggested as potential contributors *(**12**,*  *49**)*. To determine whether these ligases regulate those two UFD model substrates, we conducted an siRNA screen (Fig. 3A and Fig. S1). We excluded HUWE1 from this screen because its knockdown consistently caused significant cell death in our hands. First, we confirmed siRNA knockdown efficiency using DIA-MS analysis of total cell lysates (Fig. 3B and Table S1C). WB analysis revealed that knockdown of UBR4, KCMF1, UBE4B or TRIP12 all resulted in varying degrees of accumulation of both Ub-GFP and Ub-GFP-tail, with UBR4 exhibiting the strongest influence on substrate stabilization, followed by KCMF1 (Fig. 3A and C, Table S1D). Recent studies have reported that UBR4 and KCMF1 form a complex that collaborates to extend K48-linked ubiquitin chains *(**44**,*  *45**,*  *48**)*. Supporting this, we observed the mutual dependency on stability between UBR4 and KCMF1: knockdown of UBR4 decreased KCMF1 protein levels, and conversely, knockdown of KCMF1 reduced UBR4 levels, even though KCMF1’s knockdown efficiency was relatively modest (Fig. 3B). UBR5 knockdown had no detectable effect on either substrate (Fig. S1), so we did not include it in subsequent analyses.

To further investigate how these E3 ligases affect the specific ubiquitin linkages on the substrates, we performed Ub-AQUA/PRM analysis following knockdown of the identified E3s. Our results showed that each E3 ligase had distinct impacts on the abundance of K48- and K29-linked ubiquitin chains depending on the substrates (Fig. 3D and Table S2B). To accurately determine the stoichiometry of ubiquitin chains conjugated to the substrate, each ubiquitin linkage quantified by Ub-AQUA/PRM was normalized to the amount of GFP in the immunoprecipitates quantified by MS (Fig. 3E and F, Table S2C and D). In Ub-GFP, knockdown of UBR4, KCMF1, UBE4B and TRIP12 significantly reduced both K29- and K48-linked ubiquitin chains, with UBR4 showing the most pronounced reduction (over 90%) (Fig. 3E). In contrast, for Ub-GFP-tail, knockdown of UBR4 and KCMF1 led to a substantial reduction of over 60% in K48-linked chains and up to 40% in K29-linked chains (Fig. 3F). Conversely, knockdown of UBE4B or TRIP12 showed minimal changes in either K48- or K29-linked ubiquitin chains on Ub-GFP-tail (Fig. 3F). These findings highlight the crucial role of the UBR4–KCMF1 complex in targeting UFD substrates for degradation *in vivo*, thereby validating very recent *in vitro* studies, which showed that this complex recognizes mono-ubiquitinated substrates and catalyses the extension of K48-linked ubiquitin chains *(**44**,*  *48**)*.

Previous studies have reported that TRIP12 generates K29/K48 branched ubiquitin chains by inserting K29-linked branches into K48-linked chains *(**50**,*  *51**)*. This mechanism amplifies the ubiquitin signals and facilitates the proteasomal degradation of hard-to-degrade substrates *(**12**,*  *20**,*  *50**,*  *52**)*. Consistent with this, TRIP12 knockdown led to stabilization of Ub-GFP, accompanied by a reduction in both K29- and K48-linked ubiquitin chains (Fig. 3A and C). In contrast, Ub-GFP-tail showed only a slight, non-significant stabilization. Note that in the case of Ub-GFP-tail, K29-linked chains remained unchanged by TRIP12 knockdown, suggesting the potential involvement of other K29-linkage-generating E3 ligases, such as HECTD1 and UBE3C, in its ubiquitination *(**51**,*  *53**,*  *54**)*.

Regarding UBE4B, its yeast homologue Ufd2 has been reported to cooperate with p97 and ubiquitinate UFD substrates by catalysing the formation of K48-linked chains on pre-existing K29-linked distal ubiquitin *(**12**,*  *36**,*  *55**,*  *56**)*. UBE4B knockdown led to the accumulation of both Ub-GFP and Ub-GFP-tail, though to a lesser extent than observed with other E3 ligases (Fig. 3A and C). Curiously, Ub-GFP showed a decrease in K29- and K48-linked ubiquitin chains upon UBE4B knockdown, whereas Ub-GFP-tail ubiquitination remained unchanged (Fig. 3E and F). Therefore, the precise role of UBE4B in Ub-GFP-tail degradation requires further investigation.

Integrating our results with previous findings, we propose the following model for ubiquitin chain assembly on Ub-GFP and Ub-GFP-tail (Fig. S2). In both substrates, the uncleaved ubiquitin moiety, which serves as the initial ubiquitination mark, is recognized and extended with K48-linked chains catalysed by the UBR4–KCMF1 complex *(**44**)*. For Ub-GFP, TRIP12 inserts K29 branching on the K48-linked chains, and then UBE4B catalyses K48-linked ubiquitination on the K29-linked chains, likely following unfolding by p97, thereby facilitating its subsequent proteasomal degradation *(**12**,*  *50**,*  *55**)*.

### p97 and RAD23B strongly associate with Ub-GFP

**Fig. 4:**
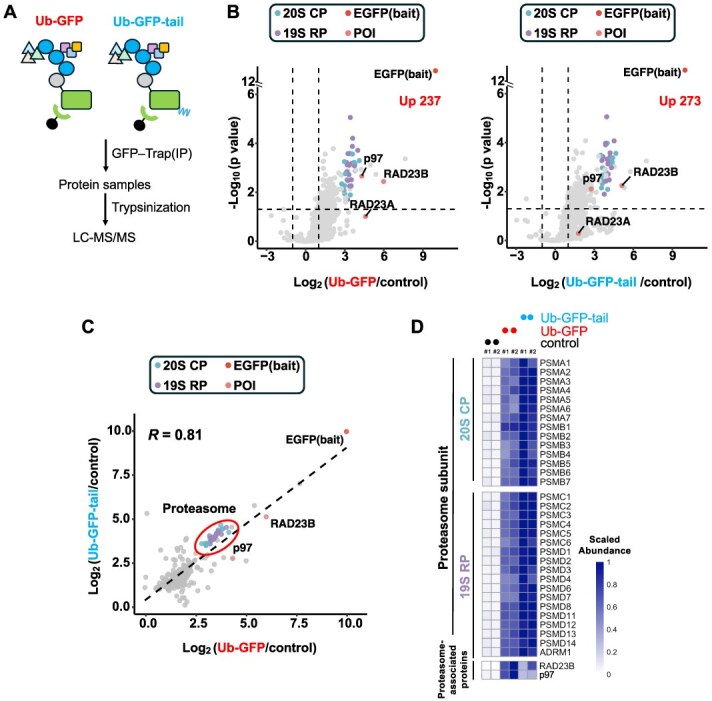
**Ub-GFP and Ub-GFP-tail exhibit distinct binding preferences for the proteasome, RAD23B and p97.** (A) Schematic of the experimental workflow. GFP-tagged Ub-GFP and Ub-GFP-tail were immunoprecipitated using GFP-Trap, followed by trypsin digestion and LC–MS/MS analysis. (B) Volcano plots showing proteins enriched in the interactomes of Ub-GFP (left) and Ub-GFP-tail (right) compared to control (log₂FC > 1, *P* < 0.05). Proteasome subunits (20S CP: blue; 19S RP: purple) and key UPS components (p97, RAD23A, RAD23B) are highlighted. (C) Scatter plot comparing the interactomes of Ub-GFP and Ub-GFP-tail. (D) Heatmap of scaled abundance of proteasome subunits and proteasome-associated proteins. Values represent scaled abundance across Ub-GFP, Ub-GFP-tail and control.

To further investigate the molecular basis for the differences in degradation pathways between Ub-GFP and Ub-GFP-tail, we performed GFP pull-down followed by MS to identify their respective interactors (Fig. 4A and B). The interactome similarity analysis revealed a high degree of correlation between the two substrates (*R* = 0.81) (Fig. 4C). However, Ub-GFP-tail showed stronger affinity for proteasome subunits (indicated by blue and purple dots), while Ub-GFP exhibited stronger association with RAD23B and p97 (Fig. 4C). A heatmap further confirmed this finding (Fig. 4D). These results suggest that the increased proteasome binding to Ub-GFP-tail, through engagement of its unstructured region, thereby reducing its dependency on p97-mediated unfolding. Among five major shuttle factors (RAD23A/B, UBQLN1/2/4), only RAD23A/B were identified in the experiment. This specificity might be explained by differences in chain-type selectivity: RAD23A/B strongly bind to K48-linked ubiquitin chains with high selectivity, whereas UBQLNs lack linkage selectivity, yet they undergo liquid–liquid phase separation with K63-linked chains *(**6**,*  *24**,*  *33**,*  *57**,*  *58**)*. Additionally, RAD23B is known to be approximately 10-fold more abundant than RAD23A in most human cells *(**33**,*  *59**,*  *60**)*. Therefore, RAD23B predominantly interacts with Ub-GFP, as observed in this study.

### Substrate structure dictates dependency on RAD23A/B for proteasomal degradation

**Fig. 5:**
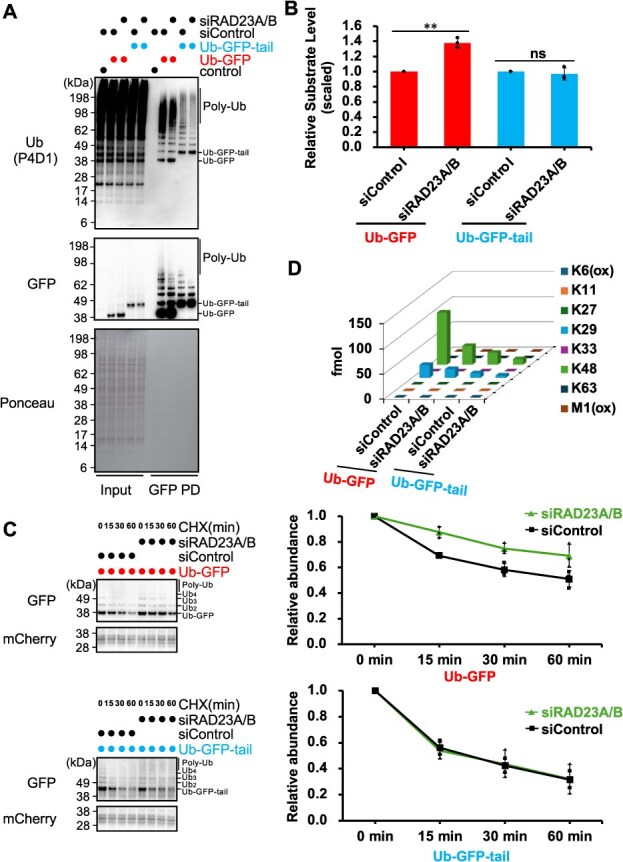
**RAD23A/B knockdown stabilizes Ub-GFP and shortens ubiquitin chains on both substrates.** (A) Western blot analysis of Ub-GFP and Ub-GFP-tail levels in HCT116 cells following siRNA-mediated knockdown of RAD23A/B. (B) Quantification of substrate levels normalized to the matched siControl (mean ± SEM, *n* = 3). Statistical significance was determined using a two-tailed one-sample *t*-test (^**^P < 0.01; ns, not significant). (C) CHX chase assays showing time-dependent degradation of Ub-GFP (top) and Ub-GFP-tail (bottom) in the presence of siRAD23A/B or siControl. mCherry served as an internal control for normalization. (D) Absolute quantification of ubiquitin chain linkages on GFP-immunoprecipitated substrates using Ub-AQUA/PRM.

Given our finding that RAD23B predominantly interacts with Ub-GFP, we next investigated the role of the shuttle factors RAD23A/B in the degradation of UFD substrates. Considering the functional redundancy of RAD23B and RAD23A, we simultaneously knocked down RAD23A and RAD23B and assessed the effects on the stability of both substrates. WB analysis showed that RAD23A/B knockdown led to a modest but clear accumulation of Ub-GFP (1.36-fold increase), whereas it had no effect on the degradation of Ub-GFP-tail (Fig. 5A and B, Table S1E). Further CHX chase analysis also confirmed that RAD23A/B knockdown significantly delayed the degradation of Ub-GFP, whereas Ub-GFP-tail degradation remained unaffected (Fig. 5C and Table S1F).

To further elucidate the impact of RAD23A/B on ubiquitin chain architecture, we performed GFP pull-down assays followed by Ub-AQUA/PRM (Fig. 5D and Table S2E). The results showed that RAD23A/B knockdown led to a marked reduction in the overall level of ubiquitin chains, concomitant with decreased amounts of K48 and K29 linkages on both Ub-GFP and Ub-GFP-tail (Fig. 5A and D). This reduction can be explained by shortened ubiquitin chain lengths, diminished branching or the loss of multiple mono-/oligo-ubiquitination events. This result is consistent with the general idea that shuttle factors protect ubiquitin chains from deubiquitinating enzymes during delivery to the 26S proteasome. Intriguingly, despite the reduction in ubiquitin chain length to a 3-mer for Ub-GFP-tail in RAD23A/B knockdown cells (*i.e.* Ub2-Ub-GFP-tail), its degradation rate remained unaffected (Fig. 5A, GFP  blot, and  C). This is consistent with previous observations that the addition of an unstructured region of Ub-GFP reduces its dependency on ubiquitin chain length for degradation, and also agreed with a recent report that K48 chain of 3-mer length is the minimal signal for proteasomal degradation *(**15**,*  *39**,*  *61**)*. Collectively, our results suggest that Ub-GFP degradation strictly depends on RAD23A/B-mediated ubiquitin shuttling, whereas Ub-GFP-tail can bypass this requirement, allowing for direct degradation by the proteasome.

## Discussion

In this study, we systematically investigated how substrate structure dictates the requirement for p97 and RAD23A/B in ubiquitin-mediated proteasomal degradation in human cells. Using two UFD substrates, Ub-GFP (a well-folded substrate) and Ub-GFP-tail (a substrate with an unstructured region), we demonstrated that Ub-GFP requires p97-mediated unfolding and RAD23A/B-dependent shuttling, whereas Ub-GFP-tail can bypass these requirements and undergo direct degradation. This study provides a refined model of UFD substrate degradation and offers additional insights into the selection of distinct UPS pathways.

Our results revealed that while both Ub-GFP and Ub-GFP-tail substrates have similar ubiquitin chain composition (approximately 80% K48-linked and 20% K29-linked chains) (Fig. 2), their degradation dependency on accessory factors varied significantly (Figs 1 and 5). Investigating the mechanisms underlying ubiquitination of these substrates, we identified the UBR4–KCMF1 complex as a critical E3 ubiquitin ligase. Our results showed that knockdown of either UBR4 or KCMF1 stabilized both Ub-GFP and Ub-GFP-tail, concurrent with a reduction of their ubiquitination levels (Fig. 3). This finding is fully consistent with, and complements, recent *in vitro* studies demonstrating that the UBR4–KCMF1 complex recognizes the monoubiquitinated substrates and extends K48-linked ubiquitin chains *(**44**,*  *48**)*, strongly suggesting its crucial role in the human UFD pathway. For Ub-GFP, we found that TRIP12 and UBE4B are also required for its ubiquitination and degradation, appearing to cooperate with the UBR4–KCMF1 complex to form complex K29/K48-linked ubiquitin chains (Fig. 3E). Intriguingly, our quantitative analysis of ubiquitin chains showed that the significant reduction in both K29 and K48 linkages upon knockdown of all four E3s (UBR4, KCMF1, TRIP12, UBE4B) implies that the formation of these complex ubiquitin chains itself antagonizes deubiquitination. While UBE4B is suggested to function after p97-mediated unfolding of ubiquitinated substrates *(**36**,*  *55**)*, its precise action point remains unknown in our experiments and requires further investigation. For Ub-GFP-tail, knockdown of TRIP12 and UBE4B results in only a slight effect on its stability and ubiquitination levels, likely because its unstructured region makes it less dependent on additional ubiquitination signals (Fig. 3F).

Additionally, our results suggest that RAD23A/B play a significant role in the degradation of well-folded substrates, collaborating with p97. Specifically, we observed that RAD23A/B and p97 strongly bind to Ub-GFP (Fig. 4) and that RAD23A/B knockdown markedly reduced ubiquitination levels on Ub-GFP (Fig. 5). This indicates that RAD23A/B not only function in substrate shuttling but also stabilize ubiquitin chains, thereby ensuring the optimal delivery of substrates to the proteasome for efficient degradation.

**Fig. 6:**
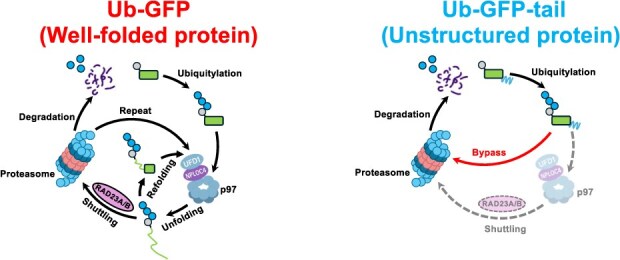
**Model for substrate structure-dependent routing in the ubiquitin–proteasome degradation pathway.** Left: For well-folded proteins, such as Ub-GFP, degradation requires p97-mediated unfolding and RAD23A/B-dependent shuttling to the proteasome. These substrates are ubiquitinated by multiple E3 ligases and subsequently unfolded by p97. If unfolding is insufficient or if the substrate spontaneously refolds, the substrate returns to p97 for additional rounds of unfolding. Following these, RAD23A/B deliver the substrates to the proteasome. Right: Substrates with unstructured regions, such as Ub-GFP-tail, can bypass p97 and RAD23A/B. This is achieved by directly engaging the proteasome and initiating degradation without the need for unfolding or shuttle factor assistance.

Based on the results above, we propose a refined model for the ubiquitin-proteasome degradation pathway of UFD substrates in human cells (Fig. 6). First, UFD substrates are recognized by the UBR4–KCMF1 complex through their ubiquitin moiety, which then catalyses the extension of K48-linked ubiquitin chains *(**44**,*  *48**)*. Subsequently, TRIP12 and UBE4B assemble K29/K48-branched ubiquitin chains *(**12**,*  *50**,*  *52**)*. During this process, the structural characteristics of the substrates might determine the threshold of the ubiquitin signal required for degradation: well-folded substrates, such as Ub-GFP, typically require more robust ubiquitination, making E3 ligases like TRIP12 and UBE4B crucial for efficient degradation. Conversely, substrates containing unstructured regions, such as Ub-GFP-tail, have reduced dependency on extensive ubiquitination.

Following ubiquitination, well-folded substrates are initially handled by p97 and then delivered to the proteasome via RAD23A/B. If the substrate’s unfolded segment is long and flexible enough to directly engage the 19S ATPase subunits of the proteasome, degradation begins, and the substrate is hydrolyzed within the 20S core particle. However, if unfolding is incomplete or if the substrate spontaneously refolds, it returns to p97 for additional unfolding *(**17**,*  *27**,*  *62**)*. This p97-proteasome bidirectional shuttling may occur repeatedly until successful degradation *(**27**)*. During the shuttling process, RAD23A/B also contribute to protecting ubiquitin chains from deubiquitinating enzymes via its two UBA domains *(**23**,*  *63*–*67**)*. This protective mechanism is especially critical for the efficient degradation of well-folded substrates.

Substrates with unstructured regions, such as Ub-GFP-tail, can also undergo p97-mediated unfolding and RAD23A/B shuttling, as our interactome analysis showed that p97 and RAD23A/B bind to Ub-GFP-tail, albeit weakly (Fig. 4). However, these steps are not strictly necessary. These substrates can bypass p97 and RAD23A/B by directly binding to the proteasomal ATPase subunits through their unstructured regions, thereby initiating degradation directly.

Collectively, structural characteristics of substrates determine their dependency on p97-mediated unfolding and RAD23A/B shuttling, thereby finely regulating the efficiency and accuracy of the ubiquitin-proteasome degradation pathway (Fig. 6). This concept holds significant biomedical implications: comprehensively understanding how substrate structure influences proteasomal degradation pathways could provide deeper insights into how UPS dysregulation contributes to diseases like neurodegenerative disorders and cancer *(**68*–*70**)*. Furthermore, our model offers new insights for the growing field of targeted protein degradation, such as PROTAC design, by highlighting the importance of considering structural properties of target substrates *(**71*–*73**)*. Specifically, targeting proteins with unstructured regions may lead to more efficient degradation by enabling direct proteasome engagement. Conversely, when selecting well-folded targets, it is crucial to consider their reliance on p97- and RAD23-mediated degradation. These structural considerations should therefore inform the design of more efficient degradation-targeting therapeutics.

## Supplementary Material

Web_Material_mvaf046
