# Supplementary material for: Substrate structure determines p97- and RAD23A/B-mediated proteasomal degradation in human cells
Source: J Biochem. 2025 Aug 11;178(5):341–53. doi: 10.1093/jb/mvaf046 (PMC12552062; doi:10.1093/jb/mvaf046)
Supplement: Web_Material_mvaf046 [file web_material_mvaf046.zip › Supplementary Figures.pdf]

SUPPLEMENTARY

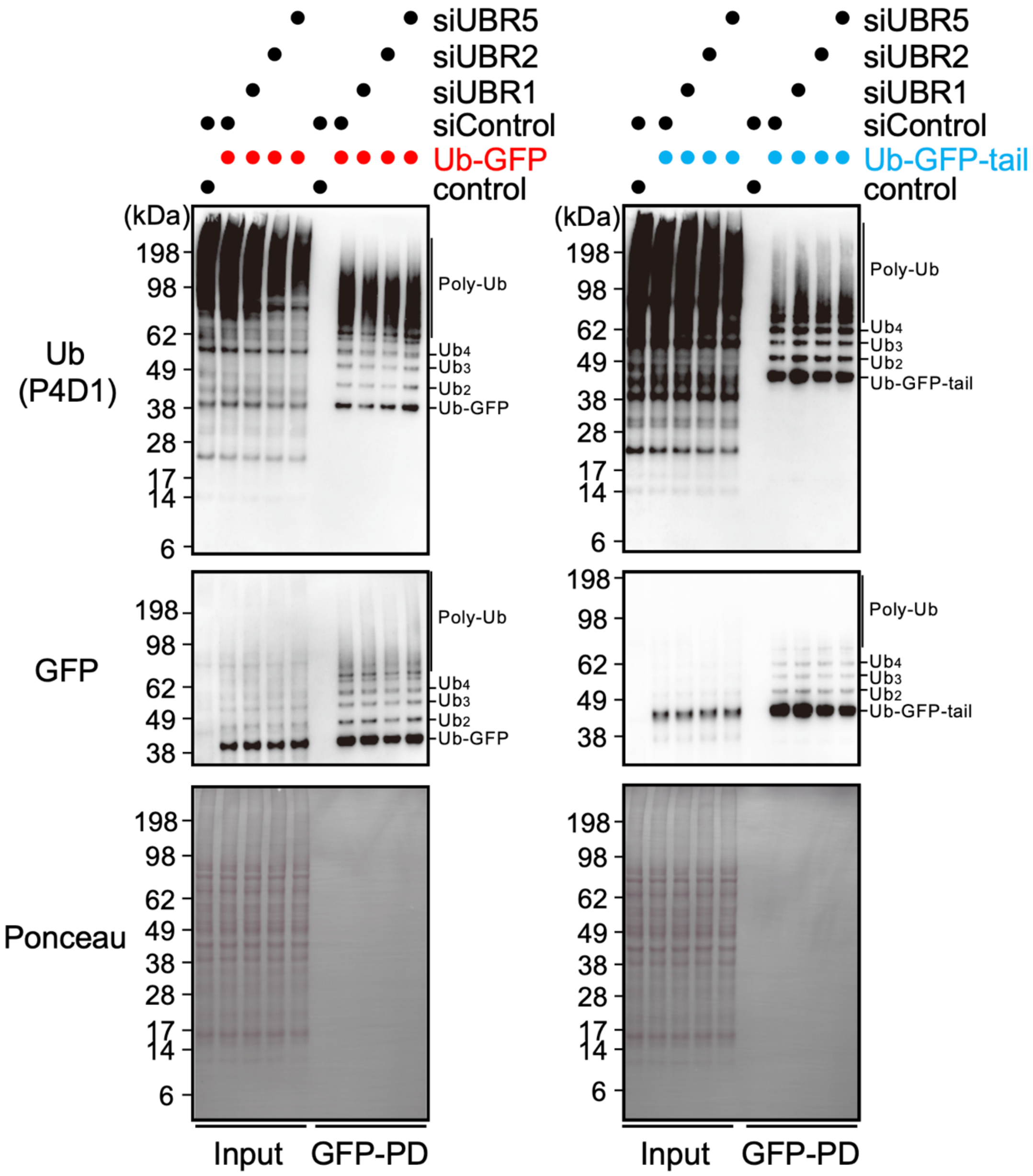

Supplementary Figure 1. Western blot analysis of Ub-GFP and Ub-GFP-tail substrates upon knockdown of UBR1, UBR2, and UBR5. Whole-cell lysates (lanes 1–6) and GFP immunoprecipitates (lanes 7–12) from HCT116 cells expressing Ub-GFP (left) or Ub-GFP-tail (right) were analyzed by Western blotting following transfection with siRNAs targeting UBR1, UBR2, and UBR5, or a non-targeting control siRNA.

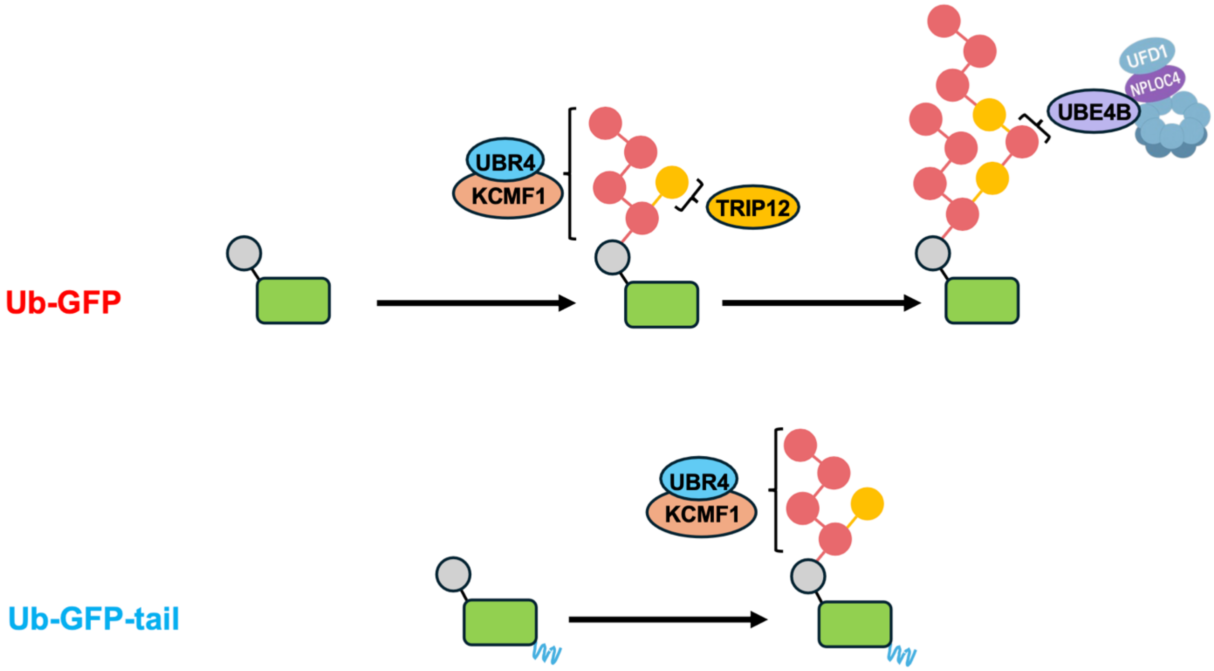

Supplementary Figure 2. Schematic model of ubiquitin chain assembly on Ub-GFP and Ub-GFP-tail. Both substrates are initially ubiquitinated by the UBR4–KCMF1 complex with K48-linked chains. In the case of Ub-GFP, TRIP12 introduces K29-linked branches, and UBE4B cooperates with p97 to extend K48-linked chains on the K29 branches.
